# Commercial vaccines used in poultry, cattle, and aquaculture: a multidirectional comparison

**DOI:** 10.3389/fvets.2023.1307585

**Published:** 2024-01-03

**Authors:** Aníbal Domínguez-Odio, Ernesto Rodríguez Martínez, Daniel Leonardo Cala Delgado

**Affiliations:** ^1^Dirección de Ciencia e Innovación, Grupo Empresarial LABIOFAM, La Habana, Cuba; ^2^Mathematics Department, Instituto de Cibernética, Matemática y Física, La Habana, Cuba; ^3^Animal Science Research Group, Universidad Cooperativa de Colombia, Bucaramanga, Colombia

**Keywords:** data, fish, veterinary vaccine, adjuvant, technological surveillance, immunoprophylaxis

## 1 Introduction

Veterinary vaccines represent a remarkable stride in enhancing animal survival and welfare. However, their benefits were not uniformly accessible to all species from the outset. In 1979 and 1998, avian and bovine species emerged as pioneers of immunization, particularly targeting *Pasteurella multocida* and *Bacillus antracis* respectively (1, 2). In contrast, fish gained immunization in 1949 against *Aeromonas salmonicida*. Commercial vaccines against *Yersinia ruckeri* and *Aliivibrio salmonicida* were not available for fish until 1976 (3–5).

While the prevention of specific pathogens in terrestrial and aquatic animals occurred at different times (6), its achievement uniformly facilitated intensive breeding for productive purposes. Consequently, the value of these species as a source of food and income increased for millions of people worldwide. The ability to pre-empt numerous infectious diseases not only bolstered profitability but also fostered safe food trade, mitigated animal suffering, reduced zoonotic infection transmission, minimized antibiotic usage, and most importantly, avoided large-scale famines across all instances (7, 8).

Currently, maintaining or expanding on these successes poses a significant challenge for the global biopharmaceutical industry. Meeting the high demand for vaccines tailored to the specificities of each pathogen and species requires robust production systems capable of producing in a stable and high yielding manner (9, 10). There are several technological platforms (traditional or modern) available for this purpose, as well as different options for obtaining effective, stable, and safe vaccines (2). The World Organization for Animal Health (WOAH) establishes international standards for vaccines in the Manual of diagnostic tests and vaccines for terrestrial and aquatic animals (mammals, birds, bees, and fish). The indications issued are written by international experts and then sent for review by scientific peers and for comments by all WOAH member countries, thus achieving consensus at the time of their adoption (11).

However, these recommendations are not sufficient to develop new vaccines; they need to be expanded with updated information on the scientific and technological advances obtained in vaccinology for each animal species. Unfortunately, this knowledge, being dispersed in different fields of science is not always visible to producers, which is why it is difficult for them to make the best decisions with a minimal risks of failure, quickly enter the market, and optimally take advantage of all available resources. Based on these facts, an investigation was conducted with the aim of comparing global trends in the manufacture and marketing of avian, bovine, and fish vaccines.

## 2 Methods

### 2.1 Source of data

A nonexperimental, observational, qualitative investigation with a descriptive scope was conducted from June to December 2022. As there was no animal involvement, ethical committee approval was not required for the analysis.

The primary source of information consisted of publicly available technical documents available on the official websites of the different veterinary biopharmaceutical companies. Only texts containing specifications of the avian, bovine, and fish vaccines marketed during 2022 (type of formulation, production technology, infectious agent, and adjuvant) were considered, whereas news and comments were excluded from the study.

### 2.2 Method of data collection

A comprehensive manual search was conducted to identify international veterinary biopharmaceutical companies engaged in the manufacturing of commercial vaccines for poultry, cattle, and fish. Companies whose headquarters were geographically located in America (United States of America, Mexico, Argentina, Chile, Colombia, Peru, Ecuador, and Uruguay), Asia (India, Russia, China, and Viet Nam), Africa (Egypt and Kenya), and Europe (Germany, Spain, France, the Netherlands, and the Czech Republic) were selected.

Initially, 29 veterinary biopharmaceutical entities were identified, with 11 ultimately included in the study (Centro Diagnóstico Veterinario S.A., ELANCO Animal Health, Laboratorios HIPRA, S.A., MEVAC, MSD Animal Health, S.L., Razi Vaccine & Serum Research Institute, Tecnovax Sanidad Animal, Vaxxinova International BV, Veterquímica, Virbac and Zoetis), totaling 587 commercial formulations. The quality of the technical information in each formulation was critically and independently assessed by two reviewers, and discrepancies were resolved through the consensus of the entire team.

#### 2.2.1 Inclusion criteria

The inclusion of each company was based on adherence to the following criteria: conducting independent research, development, and commercialization of their own vaccines, alongside maintaining an official website offering technical information about their products in english or spanish.

#### 2.2.2 Exclusion criteria

Companies that did not hold ownership of the marketed formulations, omitted detailed information on the components used, particularly adjuvants, and did not include at least one species in their official vaccine catalog were excluded from the study.

### 2.3 Variables

The characterization of each commercial formulation was based on the following variables and their categories: animal species (fish, cattle, and poultry), vaccine production technology: traditional and modern, vaccine type (inactivated, live, recombinant, subunit, DNA, and mutant strain), pathogen (bacteria, virus, mixed (bacteria+virus) and parasites), formulation (monovalent and polyvalent) and adjuvant (aluminum salts, saponin, mineral oil, other oils, and polymers).

## 3 Data analysis

To ensure systematic organization and control, the technical information of the 587 formulations provided by the 11 participating companies was registered and coded on a Microsoft Excel (2019) sheet. Descriptive statistics, including absolute and relative frequencies, were employed to characterize the comprehensive range of variables. Subsequently, groups of formulations with similar profiles were identified, and the existing associations between their attributes were assessed via multiple correspondence analyses using the R software version 4.3.1 (Bell Laboratories, USA). The variables selected to determine potential associations included animal species, vaccine production technology, formulation, pathogen and adjuvant.

## 4 Characteristics of the poultry, cattle, and aquaculture vaccines market

To characterize the international market for vaccines used in the prevention of veterinary infectious diseases in poultry, cattle and fish during the year 2022, technical information from 587 formulations developed, produced and marketed by 11 biopharmaceutical companies was used. According to the geographical location of each of these entities, the American region was the best represented with six companies (Centro Diagnóstico Veterinario S.A., ELANCO Animal Health, MSD Animal Health, Tecnovax Sanidad Animal, Veterquímica and Zoetis), followed in decreasing order by Europe with three (Laboratorios HIPRA, S.A., Vaxxinova International BV and Virbac), Asia with one (Razi Vaccine & Serum Research Institute), and Africa with one (MEVAC). The distribution by countries showed that the United States of America was the leader with three, followed by Argentina with two, while other participating countries such as Chile, Spain, the Netherlands, France, Iran, and Egypt were represented in each case by one organization.

### 4.1 Animal species

The first distinctive feature of the formulations included in the database (https://data.mendeley.com/datasets/4b26xzs5jj/1) was the dominance of avian and bovine vaccines, accounting for 42.59% (250/587) and 35.43% (208/587), respectively. Fish had the last position in the sector, accounting for 21.98% (129/587) of available commercial vaccines from 2021 to 2022. This value represents 1.94 and 1.62 times fewer commercial vaccines for poultry and cattle, respectively.

The of avian and bovine vaccines was expected, as both productive species play a vital role in the global food industry by supplying eggs, meat, milk, skins, and derivatives to millions of people (8, 12). Other factors contributing to its dominance include the need for more efficient animal protein production to meet rising global demands and an increase in markets seeking to reduce chemical residues in food (13, 14).

The significant and rising contribution of fish, particularly the *Salmo salar* species, to the global supply of high-quality protein for human consumption (15–17) seemingly did not have a sufficient effect on the use of preventive vaccines. The disproportion between birds, bovines, and fish species could be explained by multiple factors, such as the historical delay in using these formulations in clinical practice (18), limitations in understanding the pathogen–host relationships, and the variability of their habitats (19). Other important barriers that must be considered are the low yields and high costs of immunogens (5), the great diversity of rearing systems, and multiple farmed species with different vulnerabilities to infectious diseases (15, 20).

With the interest of the aquaculture industry in the immunoprevention of infectious diseases, the observed unfavorable situation may change in the coming years. The need to prevent the spread of pathogens to wild populations and reduce antibiotic use and resistance are among the motivating factors that could influence the future of these vaccines (19, 21, 22). These urgent public health concerns, combined with the economic pressure exerted by the frequent outbreaks of infectious diseases associated with intensive aquaculture methods, the transfer of fish and eggs between continents, and the monoculture of fish at very high densities (19), will encourage the process of investigation and implementation of fish vaccines, particularly the autogenous ones (23).

### 4.2 Characteristics of commercial vaccines

From another perspective, it was possible to identify a marked polarization in the use of technological platforms to manufacture commercial veterinary vaccines. The dominant vaccine types on the market were inactivated (58.9%, 346/587) and live (37.6%, 221/587), followed by recombinant vaccines (1.7%, 10/587), genetically modified virus vaccines (0.9%, 5/587), subunit vaccines (0.5%, 3/587), and DNA vaccines (0.2%, 1/587). It was also observed that the leading animal species in the market were birds (42.7%, 250/587), then cattle (35.4%, 208/587) and finally fish (21.9%, 129/587).

**Figure 1:**
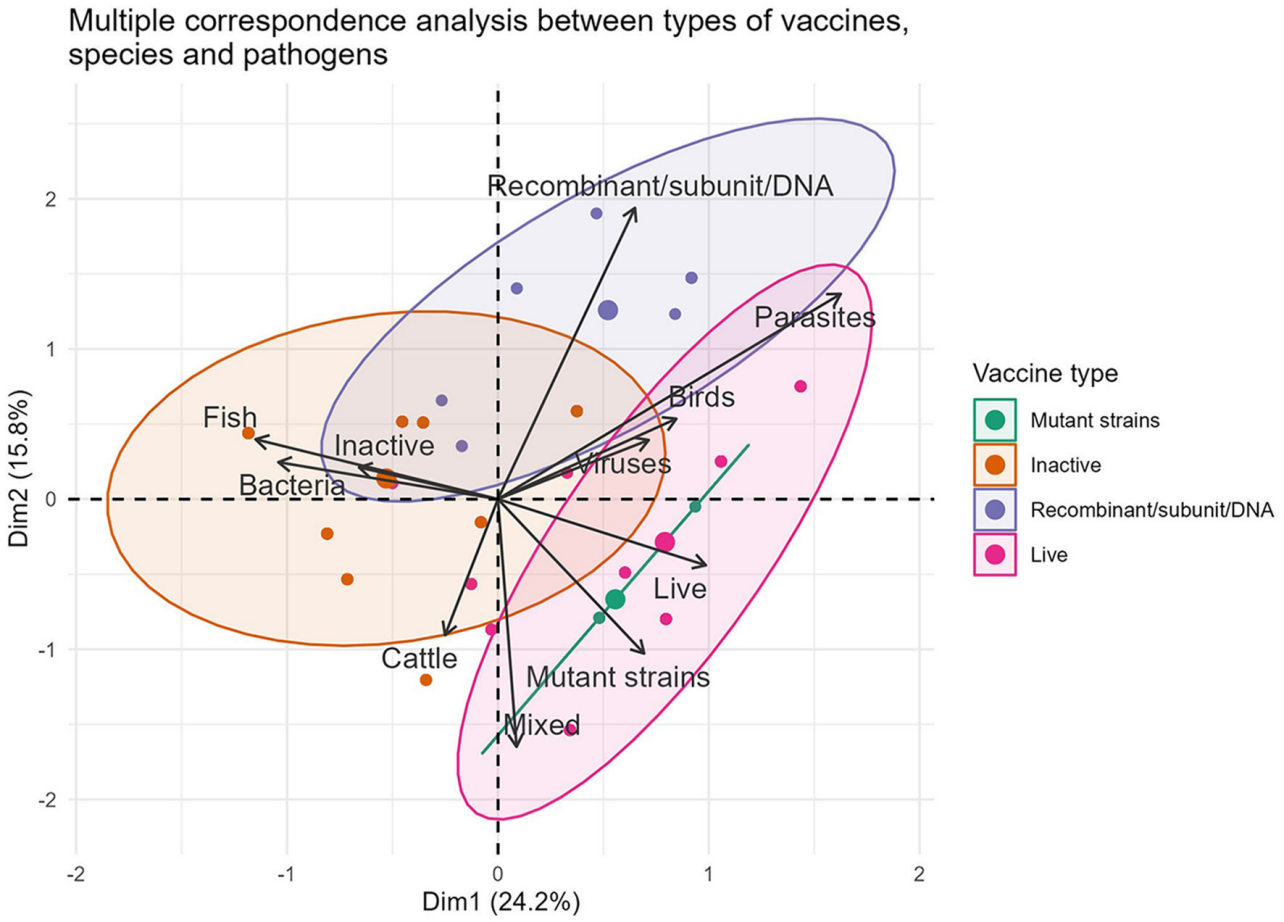
Representation of the associations between the variables vaccine type, pathogens and animal species in formulations marketed during 2022.

The traditional production methods are strongly associated with 96.59% (567/587) of formulations intended for the three forms of animal rearing. Out of these, 41.23% (242/587) are designed for avian immunization. Multiple correspondence analyses between the production technology, pathogen (formulation based on bacteria, virus and their mixed) and animal species variables identified new characteristics from the commercial point of view (Figure 1). In particular, a strong association was found between inactivated vaccine–bacteria–fish (80.62%, 104/129) and live attenuated vaccine–virus–poultry (53.60%, 134/250).

The commercial dominance of vaccines of bacterial origin and those manufactured traditionally (live and inactivated) was expected. On the one hand, it corresponds to the long history of using traditional vaccines to control infectious diseases in the main livestock farms worldwide as well as to their favorable cost/benefit ratio (24). On the other hand, it occurs at a difficult time when international reports on antibiotic-resistant bacteria are increasing and therapeutic alternatives to combat them are depleting (14, 25). The ability of certain countries to produce their own traditional vaccines using circulating local strains (23, 26), as well as the low costs of developing and manufacturing them, contribute to this strategic alliance, ensuring the profitability of local producers (7, 27). In general, all are viable solutions to protect herds against a diversity of autochthonous pathogenic strains. It also implies, in practice, eliminating excessive costs associated with the long transportation of vaccines, self-sufficiency, technological sovereignty, low sales prices per dose, and the possibility of exporting to neighboring countries (23, 28).

The scarcity of modern vaccines in the catalogs of the companies included in the study (3.24%, 19/587), indicates that much of the new scientific knowledge remains in the exploratory field, with few vaccines becoming marketable products. However, the parity in their quantity across animal species reflects that computer, genomic, and immunological breakthroughs occur in all directions, opening up new opportunities that benefit all species in a similar way (29, 30). While these vaccines' greatest advantages are antigen purification, the ability to prevent the carrier state in vaccinated animals, and the ability to differentiate between vaccinated and infected animals (10), their role in health management across the three species is unclear. They still face similar challenges as previous generation vaccines, such as dependence on the cold chain, uncertainty in predicting their potency in the field, and the need for booster doses (31–33). According to the available data, the main challenge faced by these formulations is the quest for new adjuvants that enhance the poor immunogenicity of their antigens, cause minimal reactogenicity, and can be administered orally/nasally (34).

**Figure 2:**
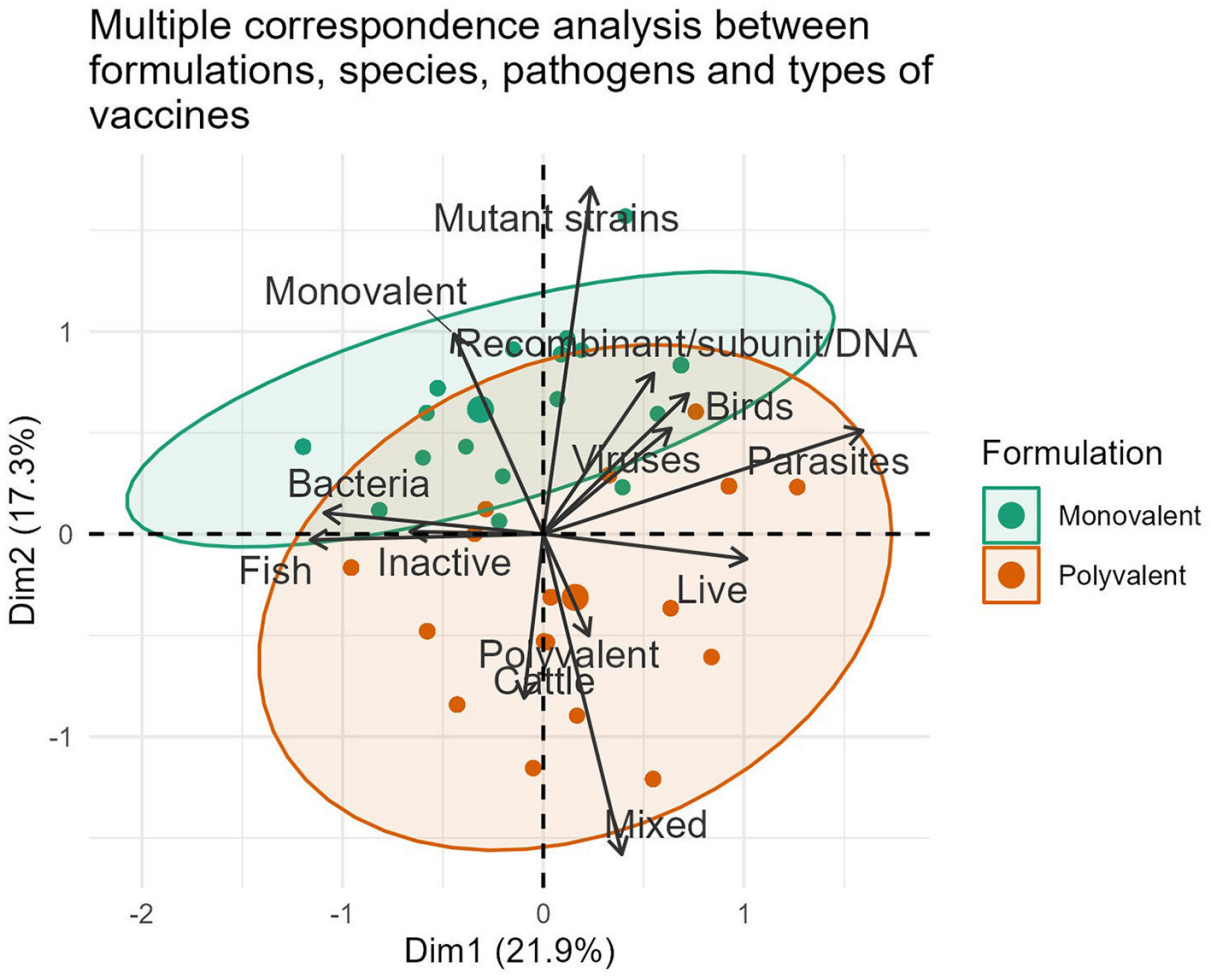
Representation of associations between the variables formulation, vaccine type, pathogen and animal species in formulations marketed during 2022.

The international market landscape for veterinary vaccines was further elucidated by identifying groups of formulations with both similar and opposite profiles (Figure 2). Polyvalent vaccines constituted the largest area in the grouping ellipse, representing 63.20% (371/587) of the formulations, especially in cattle (76.92%, 160/208) and poultry (56.00%, 140/250) vaccines. Fish continues in the last position, representing 55.04% (71/129) of the commercial polyvalent vaccines available during 2022. On the contrary, monovalent formulations were the least representative and constituted 36.80% (216/587) of the compiled data.

The strong interest of pharmaceutical companies in marketing vaccines with multiple viral or bacterial antigens in all species stems from the health needs of poultry, cattle, and aquaculture species. There are undeniable benefits to avoiding numerous diseases per dose, facilitating rapid compliance with the vaccination schedule, and increasing immunization coverage. These formulations have in common the reduction of the application, transport, and storage costs of the biologicals, as well as the reduction of stress generated in animals during vaccination handling. However, its main limitations include the possibility of antigen interference, uncertainty in determining the ideal time of administration, and difficulty in assigning responsibilities for adverse reactions. From an industrial standpoint, this segment of the market is more expensive, requires more logistics for its production, and presents challenges in developing appropriate immunological assays to determine efficacy (35, 36).

The inclusion of multiple bacterial antigens and sometimes mixed with viral antigens in traditional and modern commercial fish formulations (44.96%, 58/129), reflects the significant role these pathogens play in the massive mortalities and economic losses observed in the aquaculture sector (20, 23). Moreover, it confirms the continuous scientific and technological advances in pathogenesis, microbial genomics, and models to evaluate efficacy, which are all focused on mitigating the negative effect of these pathogens on aquaculture (5, 37).

### 4.3 Adjuvants

The analysis of the associative structure among the categories: adjuvants, vaccine production technology, vaccine type (inactivated, recombinant, subunit, and DNA), pathogen [bacteria, virus, mixed (bacteria+virus), and parasites], formulations and animal species was equally interesting and required the processing of 61.33% (360/587) of the available data. The significant closeness of the following categories was demonstrated: aluminum compounds–bovine (25.00%, 52/208) and mineral oil–fish (53.49%, 69/129). This proximity indicator, along with their positions far from the center point, allows us to infer that there is a strong relationship in all cases. The negative associations between bovine vaccines–mineral oil (3.36%, 7/208), as well as between avian vaccines–aluminum compounds (1.60%, 4/250), were also relevant.

The preference for aluminum compounds in bovine vaccines and mineral oil in fish vaccines demonstrates that global veterinary vaccine manufacturers prioritize low-cost co-stimulatants, ease of acquisition, and confidence in their safety and efficacy (38). This tendency is due to the low average sales prices of veterinary vaccines on the global market, which generate income 30 times lower than human vaccines. Such a situation necessitates the implementation of adjuvant business strategies with fewer resources, despite the complexity and variety of the hosts and pathogens (10, 39). This should not be interpreted as a lack of interest in the development of new adjuvants by the veterinary biopharmaceutical industry, rather, it is an important consideration for researchers if they intend to generalize the new molecules in the veterinary market (40).

Aluminum hydroxide and mineral oil (liquid paraffin) are not ideal adjuvants. However, they play important roles from an industrial standpoint, reducing the amount of antigen per formulation and prolonging antigen presentation to the immune system through the formation of deposits. Both have the same drawbacks that come with their use, such as adverse reactions (inflammation) localized or not at the injection site (41). Thus, the main issue when using them in veterinary vaccines is balancing their respective adjuvants and reactogenicities.

While aluminum compounds have over 70 years of clinical use in both animals and humans, known mechanisms of action, the ability to combine different antigens, an acceptable degree of safety and stability, a known chemical structure, easy preparation, and a low production cost, they are considered the reference for other adjuvants under development (42–44). Based on these data, the predominance of aluminum hydroxide in vaccine formulations for cattle (41, 45) but not for birds is evident where low antibody titers have been reported, even when dealing with inactivated vaccines against extracellular pathogens such as *Pasteurella multocida* (46).

In particular, mineral oil and non-mineral oil adjuvants have been shown to have an excellent capacity to prolong antigen release at the injection site and to generate sustained and robust responses in fish, both humoral and proinflammatory, as well as being an excellent platform for obtaining polyvalent and mixed vaccines (47). These abilities are important when formulating vaccines with antigens of weak immunogenicity (48, 49), but they might cause undesirable side effects such as tissue inflammation, melanization and adhesions between internal organs or between the organs and the peritoneal wall, necrosis and reduced growth (47, 50).

In summary, despite the absence of data from some veterinary biopharmaceutical companies such as the Japanese (DS Pharma Animal Health Co., Ltd., Nisseiken Co., Ltd. and Nippon Zenyaku Kogyo Co., Ltd.), a traditional and widespread manufacturing approach dominates on a global scale for commercial vaccines targeting poultry, cattle, and aquaculture. Traditional adjuvants (aluminum–based mineral salts and mineral oil), particularly aluminum hydroxide, have commercial hegemony despite the existence of attractive co-stimulatory molecules in the immune system.

## Data availability statement

The original contributions presented in the study are included in the article/supplementary material, further inquiries can be directed to the corresponding author/s.

## Author contributions

AD-O: Data curation, Formal analysis, Methodology, Writing – original draft. ER: Data curation, Formal analysis, Visualization, Writing – review & editing. DC: Formal analysis, Methodology, Writing – original draft.
